# Semantic Segmentation of CT Liver Structures: A Systematic Review of Recent Trends and Bibliometric Analysis: Neural Network-based Methods for Liver Semantic Segmentation

**DOI:** 10.1007/s10916-024-02115-6

**Published:** 2024-10-14

**Authors:** Jessica C. Delmoral, João Manuel R.S. Tavares

**Affiliations:** 1 Instituto de Ciência e Inovação em Engenharia Mecânica e Engenharia Industrial, Faculdade de Engenharia, Universidade do Porto, Rua Dr. Roberto Frias, s/n, 4200-465 Porto, Portugal; 2 Instituto de Ciência e Inovação em Engenharia Mecânica e Engenharia Industrial, Departamento de Engenharia Mecânica, Faculdade de Engenharia, Universidade do Porto, Rua Dr. Roberto Frias, s/n, 4200-465 Porto, Portugal

**Keywords:** Segmentation, Tumor, Vasculature, Hepatocellular carcinoma, Medical image, Liver

## Abstract

The use of artificial intelligence (AI) in the segmentation of liver structures in medical images has become a popular research focus in the past half-decade. The performance of AI tools in screening for this task may vary widely and has been tested in the literature in various datasets. However, no scientometric report has provided a systematic overview of this scientific area. This article presents a systematic and bibliometric review of recent advances in neuronal network modeling approaches, mainly of deep learning, to outline the multiple research directions of the field in terms of algorithmic features. Therefore, a detailed systematic review of the most relevant publications addressing fully automatic semantic segmenting liver structures in Computed Tomography (CT) images in terms of algorithm modeling objective, performance benchmark, and model complexity is provided. The review suggests that fully automatic hybrid 2D and 3D networks are the top performers in the semantic segmentation of the liver. In the case of liver tumor and vasculature segmentation, fully automatic generative approaches perform best. However, the reported performance benchmark indicates that there is still much to be improved in segmenting such small structures in high-resolution abdominal CT scans.

## Introduction

The liver is one of the most important organs in the human body, and impairments to its normal function are life-threatening. Liver cancer is the sixth most diagnosed cancer and the third most frequent cause of cancer death worldwide [1]. Such diagnosis may arise as a primary tumor, i.e., occurring in the liver, and usually identified as Hepatocellular carcinoma (HCC) or as a secondary cancer due to metastasis of tumors generated in other organs.

**Fig. 1:**
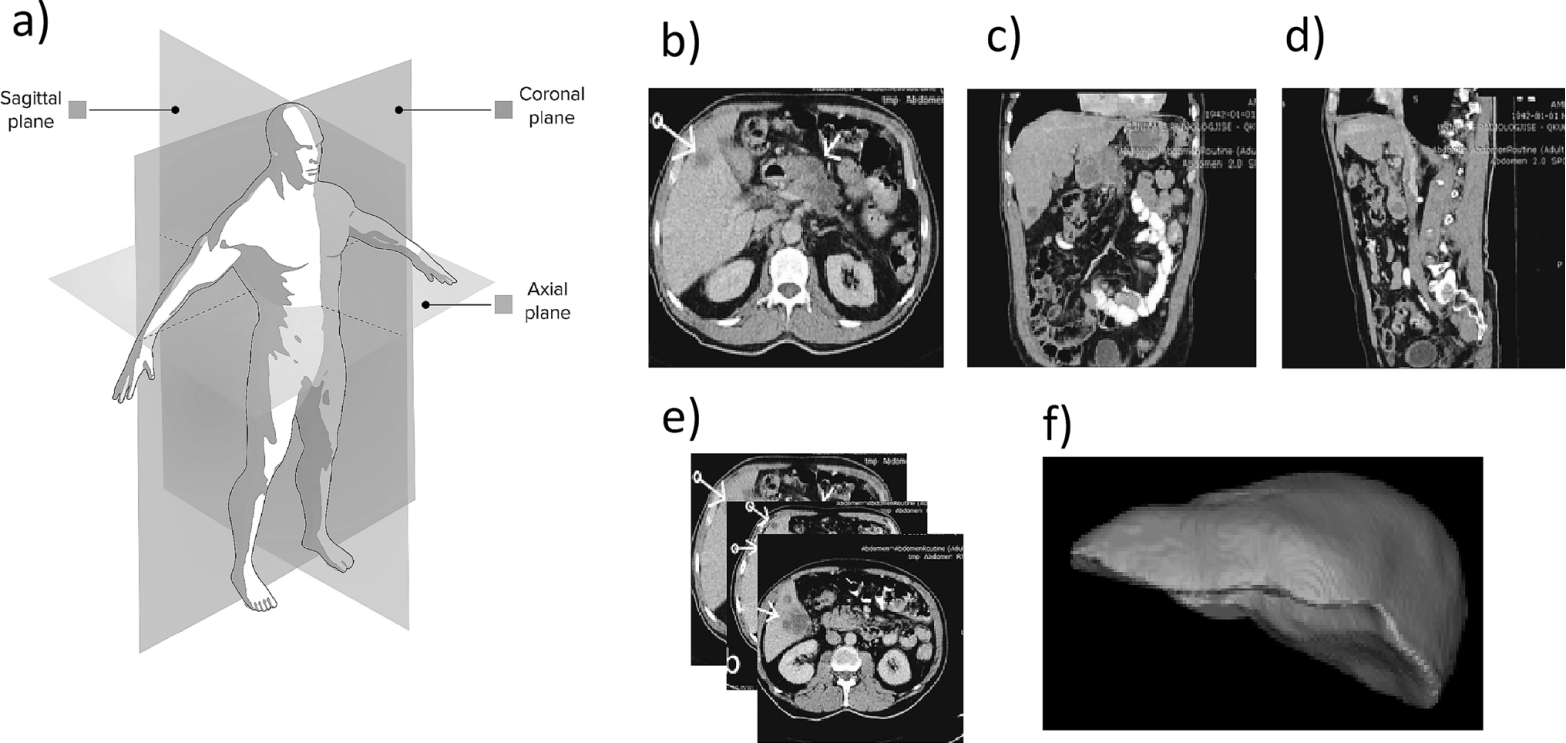
Explanation on the location and components of a CT exam: a) Depiction of the standard anatomical acquisition planes, b-d) CT slices in axial, coronal, and sagittal planes, e) A stack of CT axial slices that compose one CT exam, f) A 3D reconstruction of a liver obtained from its delineation on each axial slice of a CT exam

Medical imaging techniques are frequently used in the clinical evaluation of liver diseases to provide fairly detailed images of soft-tissue organs via a non-invasive procedure, resulting in cross-sectional images of the abdominal cavity, where the liver is located. Several imaging modalities can be used to analyze the liver in clinical practice routine, with computed tomography (CT) being one of the most frequently used, particularly in the context of liver cancer. Therefore, the segmentation of liver structures in CT images has gained increasing attention from the research community in the last decade since it represents an important step towards computer-assisted diagnosis and/or treatment planning for various hepatic diseases.

Identifying the main liver structures, mainly liver, liver tumors and vascular system, and anatomic liver regions using, for example, Couinaud’s segment mapping system [2], has been one of the main subjects of recent literature. These structures are relevant for performing several diagnoses, treatments, and follow-up tasks in clinical practice. For example, liver volume assessment is pertinent to accessing liver hepatic function. When identifying lesions in terms of number, size, and anatomic location within the liver structure, this factor becomes necessary to study the viability and approach of a resective surgery [3]. Finally, the position of the liver vasculature has to be known so that any intervention does not interfere with the blood supply of the main tissue areas [4].

In traditional medical practice, the liver structures are often manually delineated in the images under analysis. This makes training specialized professionals and developing computational systems able to perform a similar task challenging. It is also logically time-consuming, labor-intensive, and prone to subjectivity, making it inefficient and difficult to scale. Computational software that aids in this task is, therefore, highly convenient and necessary to function as a tool that complements the medical community in their practice routine.

### Liver in CT Images

Taking into consideration the vital importance of the liver for normal human function, several imaging modalities have been applied in medical practice worldwide, including Magnetic Resonance (MR), Ultrasound (US), Positron Emission Tomography (PET), and PET-CT combinations, which are used to investigate its morphological and functional structure. However, CT is the traditional and most commonly universally used imaging technique, presenting the following characteristics: i) superior spatial resolution than alternative imaging techniques, and 2) shorter acquisition times than MR or PET-CT modalities, making it the preferred and most trusted imaging modality for liver diagnostic purposes [3].

Traditional CT exams consist of the acquisition of sets of two-dimensional images, i.e., slices, that correspond to an amount of tissue, acquired consecutively over one of the three standard anatomic orientation planes: axial, coronal or sagittal (Fig. 1b-d). Usually, a set of axial CT slices, each corresponding typically to an axial width of 1.5-10 cm, represents consecutive areas of the human abdominal cavity (Fig. 1e). Any structure of interest can be delineated in each slice and finally stacked to build its three-dimensional (3D) model representation, as exemplified in Fig. 1f. Liver structures present characteristics represented by different intensity distributions in CT images. However, the process of automatic identification of these structures is especially challenging, due to the following factors:

1. The similar imaging intensities that spatially close liver organs usually present in CT images is a drawback in the segmentation process, given the low contrast between them.
2. Datasets variability is determinant in this domain in two ways: i) anatomy variability is influenced by a high range of aspects including subjects-specific ones: age, gender, ethnicity, and other congenital alterations of the abdominal region; liver-specific ones: shape, length, volume, lesion/ vasculature shape, number, and location; and ii) imaging variability influenced by acquisition process, used machine or imaging protocol, which may translate into highly different signal-to-image characteristics as well as lesion-to-liver and liver-to-background imaging intensity ranges.
3. Delineations generated for clinical practice or the development of computational algorithms depend on the human observer’s interpretation and subjectivity. Inter-observer variability is reported in several datasets [5, 6], and must be minimized to improve the robustness of the computational algorithms developed using them.

### Semantic Segmentation of Liver Structures in CT Images

The task of image segmentation consists of grouping similar regions or segments of an image as belonging to individual objects, i.e., classes. This can be achieved by three different approaches: semantic segmentation - segmenting objects belonging to a particular target class based on contextual information, often the neighbouring pixels; instance segmentation - segmenting all regions of an image as belonging to each individual object; and panoptic segmentation - segmenting each object of an image along with the objects’ classes, combining instance segmentation and semantic segmentation [7]. This review is focused on semantic segmentation since it is clearly the most explored approach in the literature related to segmenting liver structures in CT images.

In segmenting liver structures, the goal is to assign semantic labels, e.g., liver, tumor, vasculature, and background, to every pixel in the input image. The field evolution applied to liver analysis in medical images as seen in other medical oncology subjects ranges from 1) manual, semi-automatic, to the most recent automatic methods, and 2) image processing, graph-based, to the most recent neuronal network-based methods [3, 8]. The fully automated segmentation of liver structures and the appropriate generalization capacity of the computational methods remain the central challenges in this field [9]. Profound novelty in segmentation algorithms emerged only in the last half-decade with the advance of neuronal networks, particularly deep learning networks. To the best of our knowledge, no study has systematically built a detailed map of the neuronal network architectural research lines.

The remainder of this article is organized as follows. Section “Methods” describes the methodology applied in developing the current study, including extracting the relevant literature to be analysed, and presents its bibliometric analysis based on quantitative descriptors and a critical analysis of its evolution across the analysed period. Section “Review of Liver Semantic Segmentation Research Directions” presents a systematic review of the most relevant filtered literature, analysing the different research directions and scientific questions. Section “Discussion” discusses the surveyed literature, Section “Current Limitations and Future Directions” outlines future directions, and, finally, Section “Conclusion” concludes the article.

## Methods

The current study intended to review the literature of the last five years, mainly published between January 1, 2019 and December 31, 2023. This period was chosen for two main reasons: i) important public datasets, from 2017-2019/2021, became well established in the medical image field, serving as a baseline for an extensive benchmark of proposed methods by researchers worldwide, in similarity to what has happened in other medical segmentation tasks [10]; and ii) The neuronal networks field, for semantic segmentation, suffered profound advancements in terms of research novelty, focusing on segmentation of structures in medical images. Towards this end, a systematic review was conducted that intends to detail the state of the art in terms of a) a descriptive review - detailing proposed methodologies, findings, trends of research, and extracted conclusions, b) an integrative review - using critical thinking to identify open research questions and analyse common ideas and research lines, to aid researchers to position new research in the domain; c) a bibliometric analysis - describing the literature, recurring to graph and plot quantitative representations of relevant findings.

Therefore, based on the Preferred Reporting of Items for Systematic Reviews and Meta Analyses (PRISMA) statement, specifically the rationale, objective, screening protocol, and study comparison criteria of the selected bibliography [11], and concerning the semantic segmentation of liver structures in CT images, this review seeks to answer the following research questions (RQs):

**RQ1**: What are the main sources of articles of algorithmic research on semantic segmentation of liver structures?
**RQ2**: What are the trends in medical image information extraction from CT images when analysing liver structures?
**RQ3**: What are the trends in neuronal network methods when implementing segmentation algorithms for liver structures?

In the following sections, the methodology used for conducting the present systematic review is described, including the search strategy and selection criteria to obtain the reviewed works, a description of the results of the used search strategy is given, and the bibliometric analysis of the resulting body of literature is presented.

### Search Strategy and Selection Criteria

This systematic search was conducted in Scopus, an abstract and citation database hosted by Elsevier Publishers, and Science Citation Index - Expanded (SCI-Expanded), one of the core collection databases of Web of Science (WoS). The following keywords were used to find the relevant publications: “semantic”, “segmentation” AND “liver”. Excluded unrelated terms with the NOT operator in the querying: “ultrasound”, “magnetic resonance”, “histopathology”, “MR”. To strengthen the review’s validity, the screening phase included the following exclusion criteria: non-image processing fields, multi-organ segmentation approaches, bibliography referring to non-CT-related methods, non-English written articles, patents, Letters, pre-prints, scientific reports, and narrative reviews. The most relevant published articles were selected using a selection protocol, which consisted of the following steps:

**1:** Filter the retrieved bibliography published between dates January 1, 2019, and December 31, 2023 - hence comprising the last half decade;
**2:** Selection of bibliography focused on liver semantic segmentation, including segmentation of liver, lesions, or vasculature - neuronal network-based versus others;
**3:** Analysis of the bibliography pool of the previous step and selection of the retrieved neuronal networks-based methods - by inspection of the abstracts and keywords;
**4:** Inclusion of additional studies referred in some of the selected studies to provide the reader with additional information facilitating the understanding.

A second screening step of the remainder works was performed based on the full-text content. A final inclusion criteria was then used to 1) Ensure that top cited methods are included, 2) Ensure coverage of all the methodology variations contemplated in the literature, and 3) Contemplate publications presenting comparable validation methodologies. All the data was then compiled and used to answer the established research questions.

### Search Results

After applying all the inclusion and exclusion criteria at the stage of bibliography retrieval from the used search engines, the conducted search retrieved 388 works. After duplicate removal, the resulting literature was subjected to a screening analysis step based on title and abstract, resulting in 244 selected works. This led to the body of literature in the bibliometric analysis presented in the following section. Therefore, two filtering steps were performed: a) the exclusion of all non-related leading to a total of 244 documents included in the bibliometric review, and b) the full-text analysis of the most relevant publications, in terms of citations, publisher relevance, and novelty of research directions, which led to a final set of 69 relevant publications for the years 2019 to 2023, which were included in the systematic review.

### Bibliometric Analysis

A macro analysis of the 244 publications retrieved by the criteria previously detailed is presented in this section via a quantitative evaluation to provide an overview of the knowledge domain. Table 1 presents details of the retrieved bibliography on the semantic segmentation of liver structures in CT images. A data analytics approach was used to extract factual patterns of text elements that describe each scientific publication. To this end, a word co-occurrence analysis was conducted to build clustering visualizations and identify frequently used terms. This step allowed the discovery of research trends that went down to method-specific novel techniques emerging in the research field. The results of this analysis are presented in Section “Abstract and Keywords Analysis”. Secondly, the bibliography was classified into pertinent research technique trends identified in the previous section, and pertinent visual analyses of the time evolution of the literature taking these subjects into account were performed as presented in Section “Trending Topics”. Each section is accompanied by a discussion of the findings achieved with each visualization presented.

**Table 1:** Global summary statistics of the retrieved bibliography on the semantic segmentation of liver structures in CT images

Description	Value
Documents	244
Sources (Journals, Chapters, etc.)	115
Period	2019 – 2023
Authors	654
Authors per document	3
Average citations per document	5
Average co-citations in bibliography	2

**Fig. 2:**
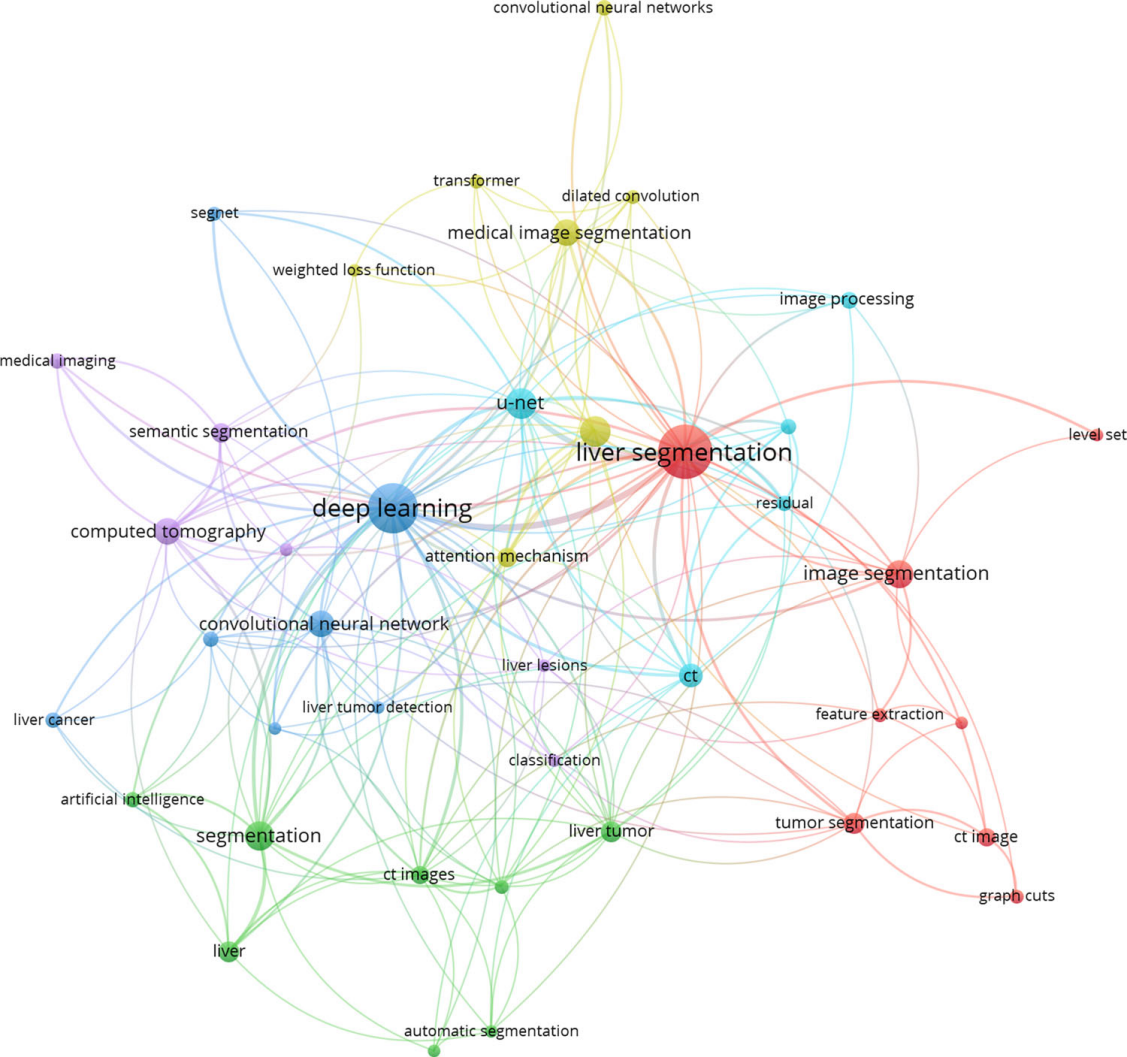
Network of co-occurring keywords generated from abstract and keyword text information from the found publications related to the semantic segmentation of liver structures in CT images published between 2019 and 2023

#### Abstract and Keywords Analysis

Keywords and abstracts are commonly considered clear and concise summaries of the context detailed in each research publication. When analysed pragmatically, they may allow one to discover groupings that affect the structure of the researched field. To this end, the following methodologies were applied to reveal research patterns: Keyword co-occurrence analysis and keyword clustering, co-citation analysis, and abstract term cluster analysis.

To build and map the knowledge domain between the topics under study, keyword co-occurrence in the research area was obtained using VOSviewer. The visualization of the keywords network was chosen to demonstrate the results of the bibliometric analysis of the literature. The output of the VOSviewer software is a distance-based map where the distance represents the strength of the relation between two knowledge topics. The item label size is directly proportional to the number of publications found where a keyword was found, and different colors represent different knowledge topics clustered by the clustering technique of the software [12]. Figure 2 presents the resulting co-occurrence map. It summarizes the field centered on the term “liver segmentation”, highlighting at the right the classical techniques of “graph cuts” and “feature extraction” methods, which still emerge frequently in the literature. However, it is possible to see on the middle-to-left side of the graph that the literature is mainly characterized by deep learning methods that vary in terms of implemented models. Regarding architecture design, the term“U-Net” appears frequently combined with one of its “residual” network variations, and combined with the term “liver”, novel proposed architecture designs such as “dilated convolution”, “transformer”, and “attention mechanism” can be found. On the other hand, works focused on cancerous lesion segmentation are scarcer in the literature and appear associated with term variations of “liver cancer”, “liver lesions” and “liver tumor”.

**Fig. 3:**
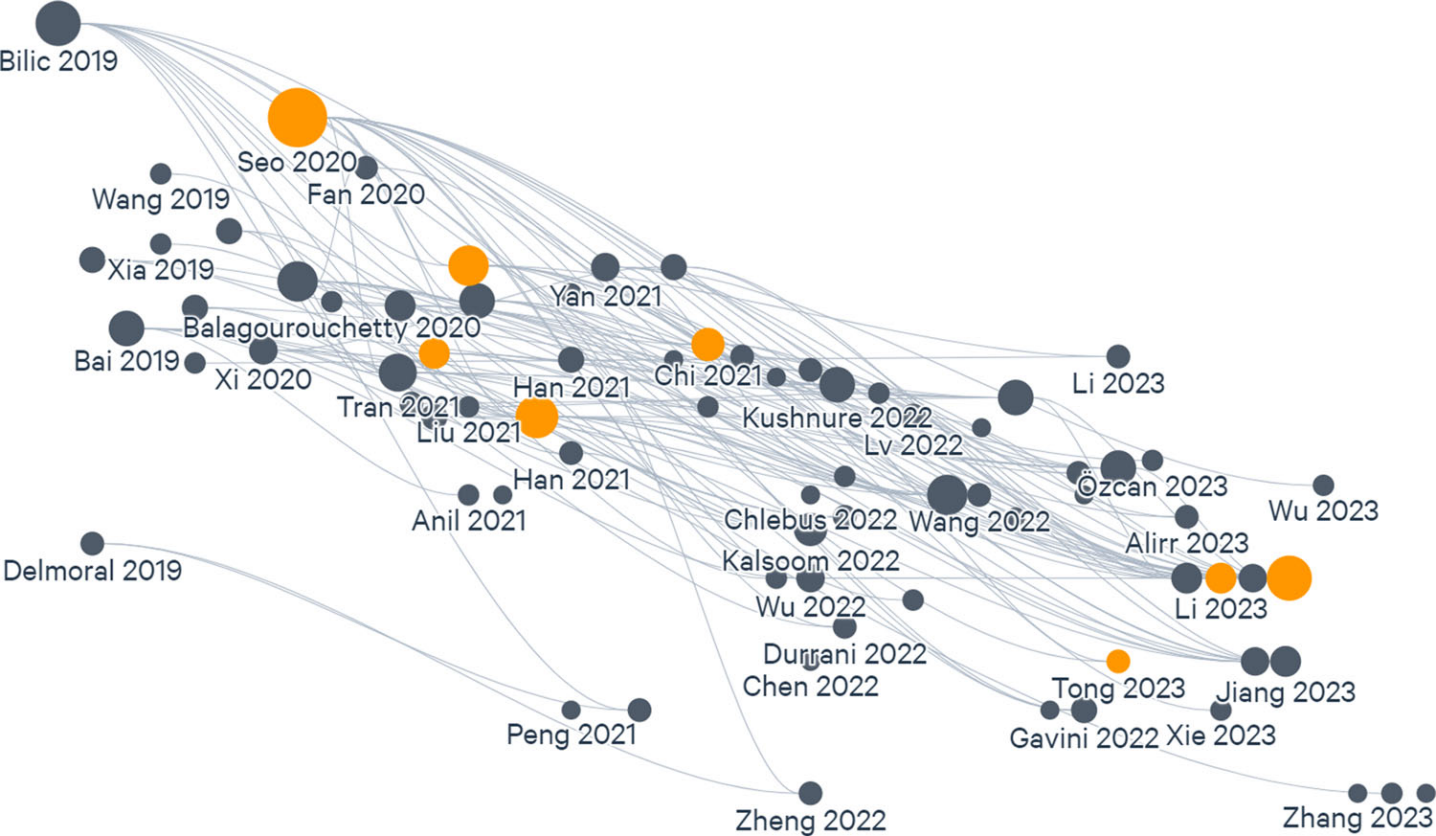
Network of co-citation patterns for publications related to the semantic segmentation of liver structures in CT images published between 2019 and 2023

The information concerning the article co-citations among the selected literature is available from the bibliographic records, and thus, the identification of the leading scientific journals, authors, and methods in the field can be mapped. The top co-cited relationships between publications can be observed in Fig. 3, where each node represents an article, the links between the authors represent co-citations, with the articles displayed in ascending date from left to right, and ascending cite count from bottom to top.

**Fig. 4:**
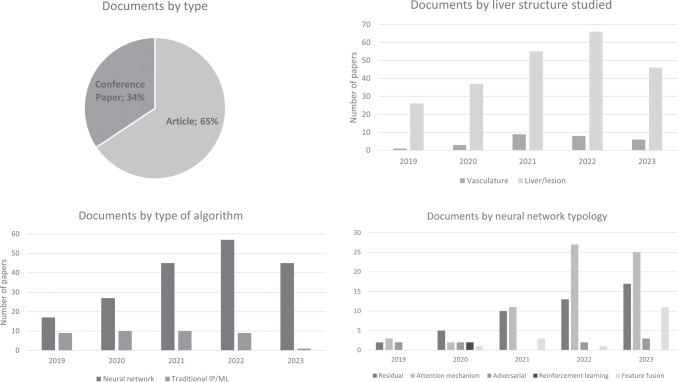
Publications published between 2019 and 2023 found in terms of (a) medium of publication (conference proceedings vs. journal article), (b) liver structure segmented (liver/tumor vs. vasculature), (c) semantic segmentation method used (“Traditional” method vs. Neuronal Network method) and (c) CNN architecture used

#### Trending Topics

Secondly, the abstract keyword co-occurrence analysis shed further insight into the relative changes of significance over time to identify trends and changes in the semantic segmentation of liver structures in CT images. To this end, an analysis that provides a time evolution of the technical methodologies that have been applied in the field under study is presented in this section.

**Table 2:** Evaluation metrics mostly found in the reviewed literature concerning the semantic segmentation of liver structures in CT images (*A*, *B* and (*d*(*s*(*A*), *S*(*B*)) are the ground truth and segmentation output, and the minimum symmetric Euclidean distance between a point $p=s(A)$ of surface *A* to surface *B*, respectively)

Metric	Description	Formula	Range
Dice	Repetition rate of the overlap between the resultant segmentation and the ground truth	$\displaystyle DSC (A, B) = \frac{2 |A \cap B|}{|A| + |B|} \times 100$	[0, 100]
VOE	Error of the volume overlap between the resultant segmentation and the ground truth	$\displaystyle VOE (A, B) = 1 - \frac{|A \cap B|}{|A \cup B|}$	[0, 1]
ASD	Mean value of the surface distance at the symmetric position between the resultant segmentation and ground truth	$\displaystyle ASD (A, B) = \frac{1}{|S(A)| + |S(B)|} \times$	$\left[ 0, \infty \right]$
		$$ \displaystyle \left( \sum _{s_A}\nolimits d(s_A, S(B)) + \sum _{s_B}\nolimits d(s_B, S(A)) \right) $$	

Figure 4a depicts the proportion between the articles published in conference proceedings and journals. In Fig. 4b, it is possible to observe the time evolution of the reviewed research regarding the articles found per year, grouped by the typology of the analysed structure. It is possible to conclude that the current research field is continuously trending, as the number of published articles has increased up to 2022, with a slight decrease in 2023. It is also possible to observe that the semantic segmentation of the liver vasculature is still an underdeveloped research topic, as a significantly smaller body of literature focuses on it. Subsequently, considering that the state-of-art can be segmented into traditional image processing and analysis and machine learning (ML) based methods, which are here referred to as “traditional methods” and “CNN methods”, an analysis of the proposed algorithm type was also performed. Thus, in Fig. 4c, it is possible to observe the time evolution of the reviewed research in terms of “traditional” versus CNN-based methods. From this figure, it is possible to conclude that the research is evolving into the usage of automated CNN-based methods, with a rising number of publications proposing novel implementations of this type. Conversely, the number of publications proposing traditional methods is decreasing.

**Table 3:** Global summary statistics of the retrieved bibliography on the semantic segmentation of liver structures in CT images (adapted from [5, 25])

Method	Description	Structure	GDC	ASD
Yuan [20]^†^	Hierarchical 2.5D FCN network	Liver	96.3	1.10
Ben-Cohen et al.	2.5D U-Net with VGG-16 backbone	Liver	96.2	1.13
Isensee et al. [22]	nnU-Net: 3D Self-adapting network + largest connected foreground post-processing	Liver	96.2	2.57
		Tumor	73.9	0.90
Xia et al. [26]^†^	An ensemble of three 3D ResNet models simultaneously co-trained	Tumor	72.1	0.89
Zou et al.	Two Cascaded U-Nets followed by hole filling post-processing	Tumor	70.2	1.19

^†^ Reference cited in retrieved bibliography that was included here to complement the reader’s understanding

Finally, looking more carefully into the neuronal network-based studies found in the literature and the implementation trends discussed in the previous section via word co-occurrence analysis, the selected bibliography can also be classified and analysed in this sense. In Fig. 4d, it is possible to observe the time evolution of the reviewed research regarding CNN architecture typology trends. It is possible to conclude that the research is evolving into the residual CNN architectures and attention mechanisms, rising steadily since 2019. Adversarial methods have appeared in the literature since 2021. More recently, the first applications of reinforcement learning were proposed (in 2022). The field’s evolution in terms of method architecture has changed in determinant ways in the past five years, and each of the mentioned research trends has stated important performance gains, as is outlined in the following section.

**Table 4:** Summary of the works found on liver semantic segmentation validated on the full Lits and/or 3DIrcadb test datasets (GDC - Global Dice Coefficient, DC - Dice Coefficient per Case, DL - Dice Loss, WCE - Weighted Cross-Entropy, CCA - Connected Component Analysis)

		Method							Performance		
Ref.	Year	Architecture	Pre-processing	Post-processing	2D/3D	Loss	N^param^	Test data	GDC Lits	DC 3Dircad	Inf. time (s)
Rezaei et al. [69]	2020	Ensemble-GAN			2D	Custom		70 Lits	94		
Sandfort et al. [66]	2019	CycleGAN			2D	Custom		20 3DIrcadb			
Demir et al. [70]	2022	Transformer CycleGAN			2D	Custom		Partial 70 Lits	94.3		
Chen et al. [85]	2019	Channel-UNet			2D	DL		20 3DIrcad		**98.4**	
Jiang et al. [53]	2019	AHCNet	DA+HU window		3D	Focal		20 3Dircad		95.9	
Jin et al. [50]	2020	RA-UNet	HU window		2D	DL	11	70 Lits, 20 3DIrcad	96.3	**97.7**	
Fan et al. [40]	2020	MA-Net	DA+HU window		2D	DL+WCE	19.4	70 Lits	96		
Li et al. [76]	2023	DHT-Net	DA	CCA	3D	unknown	59	70 Lits, 20 3DIrcadb	**97**	96.79	
Zhou et al. [38]	2020	UNet++			2D	DL + Focal	26	70 Lits	82.83		
Kushnure and Talbar [31]	2021	MS-UNet	DA+HU window		2D	DL		20 3DIrcad		97.13	6.2
Alalwan et al. [44]	2021	3D-DenseUNet-569	DA+HU window		3D	WCE	36	70 Lits	96.7		34–130
Chi et al. [86]	2021	X-Net	HU window+ resampling		2D	Region + contour loss		70 Lits, 5 3DIrcadb	**96.8**	96.68	**1.29**
Kushnure et al. [41]	2022	HFRU-Net	DA+HU window		2D	DL+WCE	4	70 Lits, 20 3DIrcadb	95.0	97.3	
Novikov et al. [63]	2019	Sensor3D	HU window		3D	DL		20 3DIrcad		95.9	

The best values found are in bold

## Review of Liver Semantic Segmentation Research Directions

The general framework of CNN methods follows three main steps: i) pre-processing of the input medical images, where various operations are performed on the images to improve their quality and variability, including resizing and normalization to reduce intensity variation, augmentation to avoid class biases and overfitting issues by generating more training samples, and removal of irrelevant artefacts or noise from the data; ii) implementation of the proposed CNN model; and, an optional step, iii) post-processing of the output probability map of label assignment to refine the final segmentation results. The present review is focused on the different scientific propositions found in the literature to solve each of the three steps of this framework.

A total of 69 articles of relevant literature were surveyed. The most used performance metrics found in the studied literature concerning the semantic segmentation of liver structures in CT images, are detailed in Tables 2 and 3. A summary of the selected and studied articles, as to the segmented structure, proposed model, dataset dimension, pre- and post-processing methods, optimization loss, and performance evaluation is given in Tables 4, 5, 6, 7. The architectures proposed are comprehensively surveyed in the following subsections categorized by the used network architecture modelling approach: U-Net architectures, other network designs, Generative models, uncertainty estimation, and interpretability methods.

**Table 5:** Summary of the works found on liver semantic segmentation validated on partial Lits and/or 3DIrcadb test datasets (GDC - Global Dice Coefficient, DC - Dice Coefficient per Case, DA - Data Augmentation, HU - Hounsfield Units, CCA - Connected Component Analysis, DL - Dice Loss, WCE - Weighted Cross-Entropy, BJPM - Biplane Joint Probability Map)

		Method							Performance		
Ref.	Year	Architecture	Pre-processing	Post-processing	2D/3D	Loss	N^param^	Test data	GDC Lits	DC 3Dircad	Inf. time (s)
Sandfort et al. [66]	2020	Modified U-Net	HU window		2D	DL+WCE	4	35 Lits, 5 3DIrcadb	97.51	96.01	1.14
Lei et al. [87]	2021	DefED-Net	HU window	CCA	2D	DL+WCE	14.5	30 Lits, 5 3Dircadb	96.3	96.6	
Lv et al. [32]	2022	RIU-Net	DA window		2D	DL+WCE	2.39	15 Lits, 20 3DIrcad CV	97.72	96.71	129-299
Lv et al. [88]	2022	DA-Net			2D	Tversky	6.4	15 Lits, 10 3DIrcad	95.8	98.17	
Zhou et al. [57]	2023	MCFA-UNet	HU window	CCA + Hole filling	3D	Tversky		20 3Dircadb, 70 Lits, 48 slices per scan	95.5	98.1	
Li et al. [54]	2020	ANU-Net	HU window		2D	DL+WCE	8.9	13 Lits	98.15		6.5
Khan et al. [75]	2022	RMS-Net			2D	DL+WCE		20 Lits, 3 3DIrcadb	97.38	97.31	
Bogoi and Udrea [89]	2022	UNeXt			2D	Focal	4.09	20 Lits		95.7	21.73
Wang et al. [52]	2023	MAD-UNet	HU window		3D	DL+WCE		15 Lits, 8 3DIrcadb	97.27	96.91	49.68
Zhang et al. [90]	2023	MSAA-Net	HU window + HE		2.5D	DL+WCE	52	20 3Dircadb, 128 slices		98.29	
Li et al. [55]	2023	Eres-UNet++	DA		2D	Dice		13 Lits	95.8		
Chen et al. [59]	2023	DRAUNet	HU window	BJPM	2.5D	DL	2.6	30 Lits	97.85	97.41	
Ma et al. [91]	2023	MDAU-Net			2D	Tversky		$\tilde{2}6$ Lits	94.3		
Sabir et al. [29]	2022	RES-UNet	DA + HU window		2D	WCE	14.32	Synthetic and real data		97.81	
Devidas et al. [92]	2023	LiM-Net	HU window		2D	DL+Focal	7.5	20 Lits, 10 3Dircad	96.3	97.3

**Table 6:** Summary of the works found on liver tumor semantic segmentation validated on full Lits and/or 3DIrcadb test datasets (GDC - Global Dice Coefficient, DC - Dice Coefficient per Case, DA - Data Augmentation, HU - Hounsfield Units, DL - Dice Loss, WCE - Weighted Cross-Entropy)

		Method							Performance		
Ref.	Year	Architecture	Pre-processing	Post-processing	2D/3D	Loss	N^param^	Test data	GDC Lits	DC 3Dircad	Inf. time (s)
Pang et al. [67]	2020	CTumorGAN			2D	Custom		70 Lits	80.19		
Rezaei et al. [69]	2020	Ensemble-GAN			2D	Custom		70 Lits	84		
Sandfort et al. [66]	2019	CycleGAN			2D	Custom		20 3DIrcad			
Chen et al. [85]	2019	Channel-UNet			2D	Dice		20 3DIrcad		**94.7**	
Jiang et al. [53]	2019	AHCNet	DA+ HU window		3D	Focal		20 3Dircad		66.8	1.35
Jin et al. [50]	2020	RA-UNet	HU window		2D	Dice	4	70 Lits, 20 3DIrcad	79.5	83	**0.25**
Fan et al. [40]	2020	MA-Net	DA+ HU window		2D	Dice+WCE	19.4	70 Lits	74.9		
Wang et al. [36]	2021	HDA-ResUNet	HU window		2D	WCE	16	70 Lits	65.3		
Li et al. [76]	2023	DHT-Net	DA	CCA	3D	–	59	20 3Dircadb, 70 Lits	**85**	77.87	
Liu et al. [93]	2021	SFF-Net			2D	CE (Custom)		70 Lits	76.4		
Kushnure and Talbar [31]	2021	MS-UNet	DA+ HU window		2D	Dice		20 3DIrcad		**84.15**	6.2
Alalwan et al. [44]	2021	3D-DenseUNet-569	DA+ HU window		3D	WCE	36	70 Lits	**80.7**		34–130
Chi et al. [86]	2021	X-Net	HU window+ resampling		2D	Region + contour loss		70 Lits	76.4	69.11	**1.29**
Kushnure et al. [41]	2022	HFRU-Net	DA+ HU window		2D	Dice+WCE	4	70 Lits, 20 3DIrcadb	61.4	77.9	
Chen et al. [85]	2019	FED-Net	DA+HU window		2D	Dice		70 Lits		76.6	

The best values found are in bold

**Table 7:** Summary of the works found on liver tumor semantic segmentation validated on partial Lits and/or 3DIrcadb test datasets (GDC - Global Dice Coefficient, DC - Dice Coefficient per Case, DA - Data Augmentation, HU - Hounsfield Units, DL - Dice Loss, WCE - Weighted Cross-Entropy)

		Method							Performance		
Ref.	Year	Architecture	Pre-processing	Post-processing	2D/3D	Loss	N^params^	Test data	GDC Lits	DC 3Dircad	Inf. time (s)
Lv et al. [32]	2022	RIU-Net	DA window		2D	DL+WCE	2.39	15 Lits, 20 3DIrcad CV	73.79	76.55	129–299
Seo et al. [94]	2020	Modified U-Net	HU window		2D	DL+WCE	4	35 Lits	89.72	68.14	1.14
Lei et al. [95]	2021	DefED-Net	HU window	CCA	2D	DL+WCE	14.5	30 Lits, 5 3Dircadb	87.52	66.25	
Kalsoom et al. [96]	2022	DeepLbV3 +WGAN	HU window		2D	Custom		Partial 20 3Dircadb		81	
Özcan et al. [82]	2023	AIM-UNet	DA + HU window	Thres.	2D	CE		618 Lits slices, 16 3DIrcadb	95.77	65.5	
Giannou et al. [97]	2022	En-ResUNet			2D	DL+WCE		Random 3Dircad		78	
Khan et al. [75]	2022	RMS-Net			2D	DL+WCE		20 Lits, 3 3DIrcadb	86.7	91.92	
Zhang et al. [90]	2023	MSAA-Net	HU window + HE		2.5D	DL+WCE		128 slices			
Li et al. [55]	2023	Eres-UNet++	DA		2D	DL		13 Lits	89.3		
Wang et al. [60]	2023	CPAD-Net	HU window		2.5D	DL	11	35 Lits, 5 3Dircad	74.2	73.7	
Chen et al. [49]	2023	MS-FANet	HU window		2D	DL+WCE	3.6	16 Lits, 20 3Dircard	85.3	81.2	16.9
Ma et al. [91]	2023	MDAU-Net			2D	Tversky		$\tilde{2}6$ Lits	83.9		

### Public Datasets - Baseline Data

Public datasets are the main drivers of novelty in segmentation methods of the liver, tumor lesions, and/or vessels in CT images. Many of these datasets emerged in the context of conference challenges and consist of image databases with ground truth delineations of the target structures. Therefore, the found datasets consist of datasets related to conference challenges that were published between the years 2007 to 2021, and other public datasets published between 2022 and 2023:

- SLIVER07 challenge - held in MICCAI 2007 conference, comprises 30 abdominal CT scans, accompanied by corresponding ground truth liver delineations, for automated segmentation [13].
- LTSC’08 challenge - held in MICCAI 2008 conference, consists of 30 abdominal CT scans, accompanied by corresponding ground truth liver lesion delineations, for automated segmentation [14].
- VISCERAL challenge - launched in 2015, provides 60 scans per two imaging modalities, MRI and CT, for multi-organ anatomical segmentation and landmark detection, including the liver [15].
- 3Dircadb - 3D Image Reconstruction for Comparison of Algorithm Database (3D-IRCADb) is a well-established database of several organ imaging scans with corresponding segmentation delineations, curated by the French Institute of Digestive Cancer Treatment. Released in 2012, provides 20 abdominal CT scans of liver and vessel annotations [16].
- Lits challenge - dataset held in competitions ISBI 2017, MICCAI 2017, and MSD 2018 (liver challenge) conferences, composed of i) 131 abdominal CT scans for training, accompanied by corresponding ground truth liver and lesion delineations; and ii) 70 abdominal CT scans manually annotated by four radiologists, for test performance evaluation in an online platform [5].
- CHAOS challenge - launched in 2021, provides 40 CT scans and 120 MRI scans for multi-organ anatomical segmentation, including the liver [17].
- Medical Segmentation Decathlon, MSD-Task08-HepaticVessel - containing 443 contrast-enhanced portal venous phase CT cases, where annotation were semi-automatically segmented, following expert adjustment [18]^†^.
- Liver Vessel Segmentation, LiVs - published in 2023, consists of 532 CT scans with liver vessel annotations delineated by three senior medical imaging experts. The resultant ground truths were generated by majority voting [19]^†^.

An extended, detailed description of the segmentation challenges and datasets can be found in [5]. The top three performer methods evaluated on the Lits dataset are summarized in Table 3 to establish the baseline performance:

**Liver Segmentation** The competition winner method consists of a 2.5D FCN network encoder-decoder with no skip connections that processed 3D CT scans slice-by-slice, receiving inputs of three stacked consecutive slices as additional context [20]. The second top performer method comprises a 2.5D FCN network using a VGG-16 as the encoder backbone and 3-slice inputs [21]. The third top performer, tied with the previous method in terms of Dice coefficient performance, was presented by Isensee et al., proving the top performance of 3D CNN. The method consists of a cascade of U-Nets: two models applied sequentially, where the first operates on down-sampled image slices to find the ROI localization, and the second is trained to refine the segmentation maps at full resolution [22].

**Liver Tumor Segmentation** The top three performing methods of CNN-methods coupled with a refinement post-processor. The tumor segmentation winner method consists of a cascade of 2D residual U-Nets, post-processed with a hole-filling algorithm [5]. The second top performer method includes a hybrid network coupling 2D and 3D U-Net convolutional paths, post-processed with the largest connected component algorithm [23]. The third top performer was proposed by Chlebus et al. and comprises a 2D U-net post-processed with a tumor pixel candidate classifier [24].

The results obtained at the benchmark allow one to perceive that liver semantic segmentation is a subject with well-established performance, solved with fully automated and fully CNN-based methods. In the case of the semantic segmentation of liver lesions in CT images, it became accepted that hybrid methods, i.e., CNN-based methods coupled with a secondary refinement algorithm, are the top performers. Regarding the smaller liver structures, their segmentation remained open to significant improvements. Several authors identify that the segmentation of lesions still suffered difficulties in CT images presenting i) fuzzy boundaries, ii) different structure sizes, and iii) low soft tissue contrast. These issues are the main factors that hinder the segmentation results and were not solved by the base U-Net architectures.

### U-net Architectures

The U-net architecture is the most explored type of CNN implementation in the reviewed literature published in 2019 to 2023, presenting significant performance improvements on the available datasets [5, 20–24] ^†^. A U-net structure, in its original form [27], consists of an encoder-decoder architecture. The encoder path consists of a sequence of convolutional and downsampling layers, followed by a decoder path and a set of activation/deconvolution and up-pooling layers. This network structure fuses higher-scale features from the encoder path to the decoder inputs via skip connections to optimize the segmentation result in the decoding path. However, this implies a loss of finer object information, especially important to identify local and smaller details of object boundaries correctly. This is particularly challenging in medical images with relatively low contrast and intensity differences between structures.

#### Feature Fusion Strategies

Techniques to help the collection of feature representations used to compute different descriptors of the original data are known as feature fusion techniques. This adds context information to be taken into account at different distances (within the sequence of network operations) and is important to detect boundaries of objects with different sizes correctly.

##### Residual Pathways

- The residual structure, firstly proposed as the Res-Net [28], improves on the previous implementation by using additional skip connections that propagate input feature maps into the output of convolutional blocks as a proposition to minimize the vanishing gradients problem that arises during optimization of convolutional networks. This implementation was validated for liver segmentation in [29] via adding residual connections to each convolutional block on both encoding and decoding paths. The authors demonstrated the superiority of their approach against previous literature on the 3D-IRCADb dataset, especially for the segmentation of the liver. Li. L. et al. also propose the Dense U-Net network, combining sets of N residual parallel paths known as ResNeXt to replace convolutional blocks, i.e., traditional U-Net [30]. Kushnure et al. proposed the MS-UNet network, combining the Res2Net block - consisting of N hierarchical residual-like connections outputting N feature maps, to replace the traditional convolutional block at both encoding and decoding paths. The method significantly increased the convolutional layers and model complexity but demonstrated superior performance than the baseline U-Net and ResNet models [31]. A seminal approach was proposed in [6], called modified U-Net (mU-Net). The authors propose an object-dependant U-Net: a) for liver segmentation, with the addition of a residual path of deconvolution and convolution to process feature maps propagated via skip connection; and b) for tumor segmentation the propagation of convolution resulting feature maps via skip connections, avoiding loss of resolution provoked by pooling operation. Several methods in the literature evaluate and confirm the positive contribution of the residual configuration in their proposed architecture via ablation studies [29, 32].

##### Dilated Convolution

- convolution operation with a wider receptive field of information while maintaining the number of computations of its traditional convolution counterpart. Used to substitute the pooling operation, as it adjusts the receptive field of the convolutional kernel without loosing the output resolution computed feature maps [33]. Delmoral et al. proposed the usage of a stack of parallel dilated convolutions, each with different dilation rates, acting as the image input feature extractor that is subsequently fed to a U-Net [34]. Liu et al. proposed a more complex model using a set of *N* dilated convolutions of input images followed by *N* U-Nets whose outputs were concatenated. The method proved to perform better than 3D CNNs when evaluated on the 3DIRCADb dataset [35]. Several methods in the literature evaluate and confirm the positive contribution of dilated convolution in the proposed architecture via ablation studies [36, 37].

##### Multi-scale Paths

- aim at enlarging the receptive field used in the feature extraction calculations by introducing new convolutional operations and/or with larger filters. However, this comes at the cost of increased model complexity, number of parameters, training, and inference time. The U-Net++ model proposes densely connected and nested decoder paths, adding skip connections and convolutional depth. The authors prove the performance superiority of U-Net++ in the segmentation of several medical image structures at the cost of a heavily more complex model [38]. The U^n^-Net model reformulates the encoding path blocks as the concatenated result of the three convolutions of each block [39]. Fan et al. proposed MSN-Net by replacing the encoder convolutional blocks with residual ones and using multi-scale fusion paths of high- and low-level resolution features [40]. More recently, Kushnure et al. proposed using the RES2Net convolutional blocks combined with the Squeeze and Excitation (SE) block to perform feature recalibration in a U-Net++ network. The authors also proved that in the proposed method, deep supervision actually damaged the resulting performance [41]. In [37], the previous method was improved by incorporating dilated versus standard convolution in a model with fewer convolutional layers and parameters than the U-Net baseline. In [42], the authors qualitatively evaluate the efficacy and limitations in liver tumor segmentation, highlighting the observed overestimation of boundaries of big tumors and underestimation of small tumors. This work again highlighted the problem of lack of resolution in small details of boundary regions that are difficult to segment successfully. Multi-scale pathways in U-Net-like architectures have also been validated for vessel segmentation. Conversely, Hao et al. proposed a dual-branched 3D U-Net, combining additional strided and dense convolution branches in both encoding and decoding paths [43].

##### Hybrid Feature Fusion

- the concept of fusing feature maps that encompass 2D and 3D convolutions. A seminal work was proposed by Li et al., the Hybrid-DenseUnet architecture, which was the top performer in liver tumor segmentation in the 2017 Lits competition with a global Dice performance of 82.4%. The method combines cascaded 2D and 3D densely connected U-Nets, encompassing intra and inter-slice information, and proved its performance advantage over others [23]. This architecture was enhanced by Alalwan et al. [44], by replacing pooling operations, i.e., by avoiding inherent resolution loss, and point-wise - 1 x 1 - convolution to merge 3D depth-wise features, and proposing a model with 5 times fewer parameters while improving the segmentation performance.

#### Attention Mechanisms

The concept of attention mechanisms aims at emulating the human visual system, in the sense that it can dynamically weight in or out specific areas of the data [45]. The mechanism of attention for image processing follows two principles: a) can model long-distance dependencies decomposing the image as multiple patches, i.e., vision transformer implementation; b) can learn a weighted mathematical representation of the inputs being convolutional-free, enabling focuses or rejection of certain parts of the input data, which is known as the self-attention mechanism [45, 46]. The literature includes implementations of attention alternatives based on spatial attention within a given channel. i.e., the feature map, different channels attention by weighing the importance of each channel component information, and point-wise attention. The reader is referred to [47]^†^ for a detailed technical explanation of each variation. It is implemented as a network block, which can be embedded inside the main CNN network, to analyze feature maps produced at different points. Attention mechanisms can be categorized as i) channel attention, ii) spatial attention, and iii) hybrid attention, i.e., integrating more than one type of attention mechanism.

Channel attention is achieved by incorporating the self-attention mechanism, most commonly done after the concatenation of feature maps at skip connection network points. This is applied to the U-Net architectures in [48–51], a modified U-Net architecture with the EfficientNetB4 and ResNet decoders in [52], to the UNet++ in [53–55].

Hybrid attention implementations, combining position-wise and channel-wise attention in [51, 56, 57], channel-wise and patch-wise attention in [58]. In the DRAUNet network, transverse and coronal inputs fed a U-Net with multi-scale residual SE blocks in the encoding path and channel attention blocks in the decoding path [59]. The CPAD-Net network combines channel and spatial attention to process every skip connection feature map outputted from the decoding path to the decoding. The authors demonstrated qualitative performance gains in terms of lesion boundary segmentation accuracy [60].

Finally, one study investigating the contribution of attention mechanisms to liver vessel segmentation was found in the literature. The contribution of spatial attention blocks at multi-scale fusion blocks in a 3D U-Net is explored in [61].

### Other Architectures

The well-known architecture Faster R-CNN, initially proposed for scene image segmentation, consists of a Region Proposal Network that produces rectangular smaller regions locating the target object. The network model that followed Faster R-CNN was the DeepLab V2, which introduced a block of Atrous Spatial Pyramid Pooling and became well-established in the literature [62]. On the 3DIRCADb dataset, the results were comparable to the ones of DRA-NET however, a two-step method is more time-consuming. Long Short-Term Memory (LSTMs) networks primarily designed to extract information from sequential data were also explored as an alternative way of computationally interpreting 3D information. In [63], the authors used stacks of three CT slices to input a bidirectional LSTM model and achieved comparable results to the state-of-the-art on the 3DIRCADb dataset. Zhang et al. proposed the LW-HCN network, a depthwise spatiotemporal transformation (DST) network, to bridge 2D and 3D features, fused by a final point-wise convolution [64]. The approach of using the two-staged 3D DST network, where the input is processed in two stages: 1 x 3 x 3 and 3 x 1 x 1 atrous convolutions, aimed to produce a lightweight model and solve the high computational complexity of direct 3D convolutions. The presented results confirmed a model parameter reduction in 10 with comparable segmentation performance to state-of-the-art. Kitrungrotsakul et al. proposed the VesselNet network for segmenting liver vessels by processing and merging coronal, sagittal, and axial plane data in multi-pathway DenseNets. The authors tested their model by processing patches of candidate vessels extracted from multi-scale filtering to build slice vesselness probability maps. The final network architecture was optimized for the 3Dircardb dataset with cross-validation and benchmarked with a 3D CNN and a single-pathway DenseNet [65].

### Generative Models and Data Augmentation

The use of generative models addresses the topic of dataset quality, and data augmentation towards improving the generated models quality. Generative Adversarial Networks (GANs) address this research line. GANs consists of two parts: a 1) discriminator *D*, which learns the latent distribution *z* of the dataset samples *x*, mapped via function *p = D(x)*, where *p* is the probability that a sample is real/synthetic; and a 2) generator *G*, which then produces new/synthetic data, via the learned mapping of *x̂ = (G(z))*, where *x̂* is the generated sample. The two parts are trained in an “adversarial setup”, i.e., using a joint loss function that converges to the generation of images “closer” to the real ones from *G*, and to an improved discrimination of real versus synthetic images from *D*. CycleGAN was proposed for image-to-image translation of abdominal CT and validated the idea of generating non-contrast-enhanced CT examples from contrast-enhanced CTs, intending to augment the dataset representability of the later category of abdominal CTs. The authors demonstrated a 68% DSC improvement on the non-contrast CT liver segmentation. This study is the most cited work of the entire literature body analysed in the current study [66].

The GANs can also combine tasks of data augmentation and segmentation. The CTumorGAN network is a version of CycleGAN customized to solve the problem of liver tumor segmentation, i.e., where the Generator portion maps input CT slices into tumor segmentation results. Proposed in [67], this network uses shape context rectification to combine information from the different output neurons and, in this way, provides the context of neighbouring tumor regions in the optimization task. In [68], the authors propose the DeepLab V3 network for the segmentation task and a generative adversarial network jointly optimized in a common weighted loss. The approach was validated using the Lits test dataset, achieving promising superior liver segmentation performance relative to other methods. In [69], an ensemble version of two consecutive U-Net-like architectures as the Generator, and *k* different discriminators that use different feature maps obtained from the Generator as input. The general architecture was optimized so that elements contribute to the objective loss, contributing to the trained Generator’s generalizability. The approach was tested for segmenting both the liver and lesion, where an improvement of the Dice coefficient of 3% and 9% for lesions and liver segmentation over CycleGAN was observed. In [70], the authors merge the self-attention mechanisms within a generative adversarial optimization architecture for liver segmentation. The Generator consists of an encoder-transformer-decoder network and a discriminator in an encoder-transformer network. The adversarial optimization of the Transformer architecture did not present significant improvements against the baseline encoder-transformer-decoder network [70]. Most recently, a benchmark of several generative adversarial network architectures to generate more liver tumor examples was presented in [71]. The authors assessed quantitatively the quality of the generated liver and lesion images using radiomics metrics, such as GLCM energy and correlation, to evaluate distribution similarity between real and synthetic lesions and compare the resulting lesions generated with different architectures: cGAN, Tub-sGAN, and PCGAN. Finally, the authors assessed the segmentation improvement of U-net architectures when trained with the synthetically generated images, demonstrating a segmentation improvement of 4.1% DSC on the top-performing method.

### Uncertainty Estimation

Active learning is optimizing data examples that maximize the model’s learning opportunity. This field has two-fold: i) to optimize model performance and generalizability, especially in real-world applications, and ii) to optimize which samples to be labeled and manage the annotation efforts. Chelubs et al. proposed a workflow to select CT slices that maximize dataset variability based on a measure of uncertainty calculated as the model predictive entropy. The effect of predictive entropy of the output softmax probability map on a 3D U-Net was analysed. The authors evaluated the model’s performance evolution by selecting new data based on model predictive entropy and proved that direct performance increases over each active workflow iteration. The authors also demonstrated how uncertainty measurements, calculated over entire CT scans, target volume, and slice-wise, can output a metric of model trustworthiness on the resulting segmentation [72]. Few-shot (FSS) models are a recent class of models that, from a small set of labeled examples, aim to extract class-wise prototypes that can be leveraged to segment similar and/or other objects in new images. Hansen et al. exploited the performance of an FSS model to uncover other abdominal classes in an abdominal CT scan in an unsupervised manner. The authors proposed the usage of a 3D encoder as an FSS model and used predictive cross-entropy as a measure of uncertainty of both the liver predictions and the extracted prototypes of other structures present in abdominal CT scans [73].

### Model Interpretability

Only one study was found in the literature exploring the interpretability of CNNs applied to segment liver tumors. Using the activation maximization DeepDreams method, the authors analyzed the influence of human-understandable lesion features on the output of a network. They gathered a set of lesion descriptors, such as lesion perimeter, sphericity, and intensity distribution features, and analyzed their effects on changes in the output activation. The analysis produced graphical representations of i) feature values that translate into greater output changes, hence enabling the calculation of a measure of sensitivity to the feature value, and ii) feature values of healthy liver regions versus lesions that translate into greater or lesser output changes, enabling the calculation of a measure of robustness to the feature value [74].

## Discussion

The research in the liver segmentation field has grown substantially over the last five years. This systematic review found 244 studies in total concerning the semantic segmentation of liver structures in CT images, 69 of which were selected for review. It is important to note that the evolution of the datasets’ quality has positively impacted this development by driving researchers’ attention to this field. As the bibliometric analysis shows, the research community has gradually decreased the attention to non-CNN methods and increased it to CNN-related methods. This evolution led to the highest performances ever published in the literature. Articles being published in recent years propose novelty in terms of 1) CNN architectures, 2) auxiliary tasks such as data augmentation - aiming at producing more robust datasets and, by extension, more robust models, uncertainty optimization - conveying information regarding the segmentation confidence, and model explanations to understand data features that contribute to the models’ decisions. The literature has been mainly focused on three structures of the liver in CT images: the liver, as a big structure; liver tumors, as small structures; and liver vasculature, as even smaller structures. Regarding the methods’ capability to solve the proposed RQs, the performance of the proposed semantic segmentation methods has greatly improved in the past five years, although at different magnitudes for each type of approach. Concerning CNN architecture novelty, current top performers are all based on a U-Net backbone, being, to date, the preferred architecture design on which researchers base their proposals.

Concerning liver semantic segmentation, the literature’s top performers reach near-perfect performance compared to ground truth annotations in the most large CT datasets available [75, 76]. This is partly due to the considerable evolution of CNN methods in segmenting “big” structures, which benefit greatly from context comparison to being correctly distinguished from other structures in the images. The top Lits dataset performer on liver segmentation improved the 2019 benchmark [5] top performer by 0.7 DSC, proposed in [76] as the DHT-Net model, presenting a 97 DSC score performance on the 70 CT test scans.

**Table 8:** Global summary statistics of the works found for liver vessels semantic segmentation validated on the 3DIrcadb dataset (GDC - Global Dice Coefficient, DC - Dice Coefficient per Case, ED - Edge Enhancing Diffusion, CED - Coherence Enhancing Diffusion, DA - Data Augmentation, MO - Morphological Operations, SVR - Small Voxel Removal, CCA - Connected Component Analysis, HU - Hounsfield Units, VPM - Vesselness Probability Mapping, CPA - Custom Probability Adjustment, DL - Dice Loss, WCE - Weighted Cross-Entropy)

		Method							Performance			
Ref.	Year	Architecture	Pre-processing	Post-processing	2D/3D	Loss	N^param^	Test data	3DIrcadb DC	LiVs DC	MSD DC	Inf. time (s)
Kitrungrotsakul et al. [65]	2019	VesselNet	VPM	CPA	3D	Negative loss	9.25	20 3DIrcad	**90.3**			19.1
Yang et al. [98]	2021	V-Net			3D	DL+WCE		8 3DIrcadb	71.5			13.5
Su et al. [80]	2021	DV-Net	DA+HU window	MO+SVR	3D	DL+WCE	47	20 3DIrcad	75.46			25.43
Hao et al. [43]	2022	HPM-Net	DA+HU window	MO+SVR+ CCA	3D	DL	5.13	6 3DIrcad	75.18			**5.65**
Yan et al. [61]	2022	LVSNet	DA		3D	DL	31	10 3DIrcad	**90.4**			9.45
Wang et al. [99]	2022	TransFusionNet	Canny edge detection		3D	DL+WCE		6 3DIrcad	89.8			
Xiao and Zhao [100]	2023	3DMT-GAN			3D	Custom		Partial LiVs		80.3		
Alirr and Rahni [101]	2023	ResDenseFCN	ED + CED		2D	DL		443 MSD images			**79**	
Tong et al. [83]	2023	SDA-UNet			3D	DL+WCE+ SmoothL1Loss	**2.5**	60 MSD cases			71.3	9.87
Gao et al. [78]	2023	LSFP-UNet			2D	Salience-Modulated loss		3Dircadb, LiVs, MSD cases	68	**80.3**	69.9	
Wu et al. [79]	2023	IBIMHAV-Net	DA	CCA	2D	weighted DL		4 3Dircadb	74.8			

The best values found are in bold

Regarding tumor segmentation, the literature’s top performers are established in the range of 85 DSC proposed by the top liver performer, the DHT-Net model [76]. This represents an increase of 12 DSC relative to the 2019 Lits top performer. Attention mechanisms have proven valuable in producing detailed lesion boundaries that were grossly over-estimated by previous methods, and many authors are proposing qualitative performance examples to validate these findings in more recent publications [77]. Liver vessels are the liver structures that have earned the least attention from the scientific community. This aspect is mainly due to the lack of annotated datasets addressing these structures and the corresponding number of available samples. However, these structures are highly pertinent when analysing the 3D anatomy of the liver for various medical treatments performed in medical practice routines. The best performing proposed vessel semantic segmentation methods have highly increased their performance relying on image pre-processing steps applied to the input of the used network [65], image filtering incorporated into the network architecture [78], or on post-processing cleaning methods [43, 79, 80]. The performance of the methods validated on the bigger datasets in the field, namely the Lits and 3DIrcadb datasets (Tables 4, 6), is comparatively the most robust and generalizable in the literature. However, methods validated in partial sub-samples of these datasets allowed comparative analysis of architecture designs (Tables 5, 7). Similarly, in the case of the vasculature segmentation benchmark, detailed in Table 8), more robust datasets surfaced only in very recent years and are still clearly under-explored in the literature. The conducted literature bibliometrics study also reinforces that the literature has changed the main research direction and the number of publications tendency in 2023, towards the segmentation of liver vasculature and lesions, where there is still space for performance improvement. Generative models have proven helpful specifically in the context of liver diagnosis in CT images, where contrast-enhanced CT images are widely represented in public datasets, but the clinical practice may often use non-contrast CT scans. This is in most cases due to patient clinical limitations to using contrast. The performance of the methods validated on the bigger datasets in the field, namely the Lits and 3DIrcadb datasets (Tables 4, 6, 8), is considered robust and generalizable. However, methods validated in partial sub-samples of these datasets allowed comparative analysis of architecture designs (Tables 5, 7). The complexity of models is an aspect that researchers must take into consideration. The objective is always to achieve the highest segmentation performance with the least model complexity. Knowledge distillation of the neural network is a research direction that addresses this goal. It should be noted the research line focuses on this complexity. For example, in [81], the authors explore the concept of knowledge distillation by distilling the learned segmentation capacity of more complex networks, teacher networks, into less complex ones, student networks, with comparable performances.

## Current Limitations and Future Directions

Most of the methods found in the literature have been applied to the public datasets published throughout the years, particularly to the MICCAI Lits 2019 and 3DIrcadb datasets. The availability of datasets for liver lesions is considerably scarce [29, 82] and liver vasculature [83]; hence, the related found works were excluded from the summary benchmark. The study performance benchmarks were divided according to the magnitude of the dataset split. The selected full Lits benchmark studies were all tested on the same 70 CT test exams. The selected full 3Dircadb studies were performed in a randomized cross-validation design. The full Lits benchmark provides the biggest test dataset used across all reviewed works; however, it is not a randomized case-control test, which implies that its generalizability in “real-world” datasets may be limited. The full 3DIrcadb benchmark provides the most robust study design across all methods; however, it is a smaller test sample. It is preferable for future studies to incorporate other collected, clinically representative datasets, including, for instance, non-contrast-enhanced or triphasic CT cases. In future works, the exploration of instance or panoptic segmentation-based approaches could be explored as an alternative to solve the problem of boundary segmentation of the smaller liver structures [84]. Generative models, uncertainty estimation, and interpretability strategies implemented to explain the built CNN models are still under-explored topics in segmenting liver structures in CT images. All the aforementioned aspects are vital in producing more robust and pertinent computer-aided detection (CADe) or computer-aided diagnosis (CADx) systems for real-world clinical scenarios.

## Conclusion

Artificial intelligence applied to medical images keeps suffering critical transformations and has gained immense attention from researchers and practitioners. The conducted bibliometric study aimed at extracting and evaluating the dissemination of novel trends in the field of liver segmentation. Although many systematic reviews and meta-analyses have already been published, this is the first bibliometric analysis of this field, where 244 scientific publications were examined using a “quantitative mapping” approach. Additionally, a comprehensive review of the most recently proposed methods, supported by a comparative performance analysis, was performed, detailing results according to the different CNN-based approaches used in the literature. Generative models, uncertainty estimation, and interpretability strategies implemented to explain the produced CNN models are still under-explored in segmenting liver structures in CT images but seem to be critical to improving the robustness and the usage of CADe/CADx systems in clinical practice.

## Data Availability

No datasets were generated or analysed during the current study.
